# Qualification and Application of a Liquid Chromatography-Quadrupole Time-of-Flight Mass Spectrometric Method for the Determination of Adalimumab in Rat Plasma

**DOI:** 10.3390/pharmaceutics10020061

**Published:** 2018-05-24

**Authors:** Yuri Park, Nahye Kim, Jangmi Choi, Min-Ho Park, Byeong ill Lee, Seok-Ho Shin, Jin-Ju Byeon, Young G. Shin

**Affiliations:** College of Pharmacy and Institute of Drug Research and Development, Chungnam National University, Daejeon 34134, Korea; yuri.park.cnu@gmail.com (Y.P.); nahye.kim.cnu@gmail.com (N.K.); jangmi.choi.cnu@gmail.com (J.C.); minho.park.cnu@gmail.com (M.-H.P.); byungill.lee.cnu@gmail.com (B.i.L.); seokho.shin.cnu@gmail.com (S.-H.S.); jinju.byeon.cnu@gmail.com (J.-J.B.)

**Keywords:** adalimumab, immunoprecipitation, liquid chromatography-quadrupole TOF MS, bioanalysis

## Abstract

A liquid chromatography–quadrupole time-of-flight (Q-TOF) mass spectrometric method was developed for early-stage research on adalimumab in rats. The method consisted of immunoprecipitation followed by tryptic digestion for sample preparation and LC-QTOF-MS/MS analysis of specific signature peptides of adalimumab in the positive ion mode using electrospray ionization. This specific signature peptide is derived from the complementarity-determining region (CDR) of adalimumab. A quadratic regression (weighted 1/concentration), with an equation y = ax^2^ + bx + c, was used to fit calibration curves over the concentration range of 1–100 μg/mL for adalimumab. The qualification run met the acceptance criteria of ±25% accuracy and precision values for quality control (QC) samples. This qualified LC-QTOF-MS/MS method was successfully applied to a pharmacokinetic study of adalimumab in rats as a case study. This LC-QTOF-MS/MS approach would be useful as a complementary method for adalimumab or its biosimilars at an early stage of research.

## 1. Introduction

Since humanized and fully human monoclonal antibodies (mAbs) were approved as therapeutic pharmaceutical products, the attention for monoclonal antibody products has been growing significantly in the global pharmaceutical market [1,2].

Therapeutic mAbs offer many advantages when compared to small-molecule drugs [3]. In general, mAbs have three main characteristics: (i) target-specific binding ability to increase or decrease an important biological effect, (ii) interaction of the constant domain with cell surface receptors that causes immune-mediated effector functions, including antibody-dependent cell-mediated cytotoxicity (ADCC), complement dependent cytotoxicity (CDC) or antibody-dependent phagocytosis; and (iii) deposition of complement on multimeric immune complexes between the mAb and the target and subsequent activation of complement-dependent cytotoxicity [2,4].

Tumor necrosis factor α (TNF-α) is an inflammatory cytokine produced by activated monocytes or macrophages [5]. Therefore, TNF-α antagonist (anti-TNF) is one of the agents that has the highest affinity for TNF-α molecules and suppresses the biological activity of TNF-α [6]. One of the well-known anti-TNF drugs is adalimumab, which consists of a fully humanized monoclonal antibody and was approved by the FDA in 2002 [7]. Adalimumab is a tetramer composed of two heavy immunoglobulin G1 (IgG1) chains and two light IgG1 chains [7]. It is currently used to treat rheumatoid arthritis, psoriasis, psoriatic arthritis, ankylosing spondylitis and Crohn’s disease [8].

Traditionally, pharmacokinetic evaluation of mAbs has been mainly performed by immunoassays such as enzyme-linked immunosorbent assays (ELISA), radioimmunoassay immunofluorescence assay and etc. [9]. Immunoassays have several advantages in terms of high sensitivity, robust performance and high throughput [2,10]. However, these methods also have several disadvantages such as non-specific binding as well as time-consuming and labor-intensive reagent development [8]. Immunoassays also often show cross-reactions with precursors of the target protein or with smaller metabolized fragments that are not suitable for early-stage mAbs bioanalysis [11].

Liquid chromatographic mass spectrometry (LC-MS/MS) is a complementary technique that can quantify not only small molecules or peptides but also proteins [12]. LC-MS/MS is accurate and precise and enables throughput analysis because this combines a robust separation technique with identification and quantification based on the molecular weights of the analytes [10]. In addition, LC-MS/MS is less matrix-dependent than ELISA [13].

The purpose of this paper is to explore an adalimumab quantification method using LC-QTOF-MS/MS and employing adalimumab’s specific signature peptides that are involved in variable regions and produced by tryptic digestion.

## 2. Materials and Methods

### 2.1. Materials

Adalimumab was purchased from Dongwon Pharmaceutical Wholesale (Deajeon, Korea). Protein A magnetic beads were purchased from Millipore Corp (Billerica, MA, USA). RapiGest surfactant was purchased from Waters Korea (Seoul, Korea). 1,4-Dithiothreitol (DTT) was purchased from Carl Roth (Karlsruhe, Germany). Iodoacetic acid (IAA) was purchased from Wako (Osaka, Japan). The sequencing grade modified trypsin was purchased from Promega (Madison, WI, USA). All other chemicals were commercial products of analytical or reagent grade and were used without further purification.

### 2.2. Preparation of Stocks, Standard (STD) and Quality Control (QC) Samples

Stock solution of adalimumab was prepared at a concentration of 5000 μg/mL in a phosphate buffer solution (PBS) containing 0.1% tween 20. The stock solution was stored at 4 °C. Stock solution of adalimumab was further diluted at a concentration 500 μg/mL in PBS containing 0.1% tween 20 as sub-stock solution. Calibration working solution was prepared by serial dilution of the sub-stock solution with PBS containing 0.1% tween 20. Then, the working solution was spiked into rat plasma to yield calibration standard concentrations of 1.0, 2.0, 5.0, 10, 20, 40, 80 and 100 μg/mL. The quality control (QC) samples with final concentrations of 2.5, 25 and 50 μg/mL were also prepared in the same manner.

### 2.3. Preparation of Sample Digests for Quantification

Each 24 μL aliquot of plasma study samples, QCs and standards (STDs) were separately mixed with 370 μL of PBS containing 0.1% tween 20 and 30 μL of magnetic bead suspension. After gentle shaking at room temperature overnight, the magnetic bead was washed using 600 μL PBS containing 0.1% tween 20 and then was washed again using 600 μL PBS. Seventy-five microliters of RapiGest and 10 μL of DTT were added to the mixture, which was incubated for 50 min at 60 °C to denature and reduce the adalimumab bound to the magnetic beads. After a 10-min incubation at room temperature (RT), 25 μL of IAA was added and the sample was incubated in dark conditions for 30 min at RT. Ten microliters of the sequencing grade-modified trypsin were added to the sample to digest the antibody. After 1 min of shaking, the sample was incubated at 37 °C overnight. Fifteen microliters of 2 N HCl were added to the sample for quenching purposes and the sample was incubated for another 30 min at 37 °C to stop the digestion. The resulting sample was centrifuged at 7000 rpm for 5 min at 4 °C and transferred into a HPLC vial.

### 2.4. Liquid Chromatography–Mass Spectrometry

The liquid chromatography–mass spectrometry system consisted of a Shimadzu CBM-20A HPLC pump controller (Shimadzu Corporation, Columbia, MD, USA), two Shimadzu LC-20AD pumps, a CTC HTS PAL autosampler (CEAP Technologies, Carrboro, NC, USA) and a quadrupole time-of-flight (Q-TOF) TripleTOF^TM^ 5600 mass spectrometer (Sciex, Foster City, CA, USA). The analytical column used for this assay was a Phenomenex Kinetex Phenyl-hexyl column (50 × 2.1 mm 2.6 μm). The mobile phase consisted of: mobile phase A, distilled and deionized water containing 0.1% formic acid; and mobile phase B, acetonitrile containing 0.1% formic acid. The gradient was as follows: from 0 min to 0.6 min, 5% B; from 0.5 min to 1.6 min by a linear gradient from 5% B to 95% B; 95% B was maintained for 0.2 min; from 1.8 min to 1.9 min by a linear gradient from 95% B to 5% B and then 5% B was maintained for 1.5 min for column re-equilibrium. The gradient was delivered at a flow rate of 0.4 mL/min and the injection volume was 10 μL.

The TOF-MS scan mass spectra and TOF-MS/MS scan mass spectra were recorded in the positive ion mode. For TOF-MS scan, *m*/*z* 100~950 with 0.2 s accumulation time was used. For TOF-MS/MS scan, the scan range was *m*/*z* 500~1000. For the quantification, doubly charged [M + 2H]^2+^ ion for the specific signature peptide APYTFGQGTK (*m*/*z* 535.4) was selected and its product ion at *m*/*z* 901.8 was used for the quantitative analysis of adalimumab. High-purity nitrogen gas was used for the nebulizer/Duospray^TM^ and curtain gases. The ESI spray voltage was set at 5500 V. The source temperature was 500 °C. The curtain gas (CUR) was 30 L/min; the auxiliary gas setting (GS1 and GS2) was 50 L/min.

### 2.5. Method Qualification and Sample Analysis Procedure

#### 2.5.1. Calibration Curve, Accuracy and Precision

Method qualification was carried out with a ‘fit-for-purpose’ approach. Quality control (QC) samples as well as standards (STDs) were used for batch acceptance. The qualification run contained duplicate calibration curves at eight concentrations and QCs at three concentrations (low, medium and high concentrations). The acceptance criterion for STDs and QCs in the qualification run was within ±25% of precision and accuracy, which is acceptable for early-stage drug discovery. Calibration curve was done by establishing a quadratic regression function, with an equation y = ax^2^ + bx + c after 1/concentration weighting. In addition, two blank plasma samples were in the set. QC samples (2.5, 25 and 50 μg/mL) were processed and analyzed three times in the same run (precision). The accuracy was calculated at each QC concentration as the ratio of the measured concentration to the nominal concentration multiplied by 100%.

#### 2.5.2. Species-Dependent Matrix Effect

For species-dependent matrix effect test, three levels of QC samples were prepared in mouse and monkey plasma. Samples were quantitated with a calibration curve prepared in rat plasma. Mean accuracy and precision were also calculated.

#### 2.5.3. Freeze and Thaw Stability

The freeze and thaw stability in rat, mouse and monkey plasma was assessed using low, medium and high QC samples (*n* = 3 at each concentration). For this study, the samples were subjected to three freeze and thaw cycles at −80 °C.

### 2.6. Software

Analyst^®^ TF Version 1.6 (Sciex, Foster City, CA, USA) operated with Windows^®^ (Microsoft) was used for instrument control and data acquisition. Peak integrations were operated by MultiQuant^®^ Version 2.1.1 (Sciex, Foster City, CA, USA). Calculations including peak area ratios, standard curve regressions, sample concentration values and descriptive statistics were calculated with MultiQuant^®^ Version 2.1.1. Pharmacokinetic calculations were performed using WinNonLin^®^ version 6.4 (Pharsight Corporation, Mountain View, CA, USA).

### 2.7. Application for a Pharmacokinetic Study in Rat

Four adult male Sprague–Dawley rats (SD, 250–300 g) were purchased from the Samtako Biokorea Co. (Gyeonggi, Korea). The animals were housed in laminar flow cages that were maintained at 22 ± 2 °C and 50–60% relative humidity. The animals were kept in these facilities for at least a week prior to the experiment and were fasted for at least 24 h before the commencement of the experiments. Rats were cared for and treated in accordance with the Guiding Principles for the Use of Animals in Toxicology adopted by the Society of Toxicology (Reston, VA, USA) and the experimental protocols were approved by the Animal Care Committee of Chungnam National University (protocol No. CNU-00560).

Plasma samples were collected from the Sprague-Dawley rats (*n* = 4) after dosing intravenously with adalimumab at 1 mg/kg. The sampling times were 0.0014, 0.0417, 0.1667, 0.25, 1, 2, 3, 4, 7, 15, 21 and 28 days. The same set of pharmacokinetic study samples was analyzed by LC-QTOF-MS/MS.

## 3. Results

### 3.1. Method Development

#### 3.1.1. Sample Preparation Method

In general, biological matrices such as plasma have highly abundant endogenous proteins such as albumin and immunoglobulins [14]. These endogenous proteins often interfere with the analysis of target mAb and decrease the sensitivity of the analyte. Therefore, a sample preparation method with minimal endogenous protein interference was necessary. To solve this problem, several approaches have been considered, such as albumin depletion kit, solid phase Protein A, and specific immunocapture [2,10,15]. In this study, the immunocapture using protein A magnetic beads was considered to be acceptable due to its selectivity as well as specificity. Plasma IgG and mAb were captured by a protein A magnetic bead and then washed out to remove other endogenous interference. After that, plasma IgG and mAb, bound to a protein A magnetic bead, were digested on-bead by the sequencing grade-modified trypsin. Trypsin-cleaved lysine and arginine residues in the amino acid sequence and various digested peptides from plasma IgG and mAbs were released into the supernatants. The supernatants were then analyzed by LC-QTOF-MS/MS for the target-specific signature peptide of adalimumab.

Generally, it would be ideal if trypsin digestion was carried out after acid dissociation and elution. However, one big challenge of this approach would be recovery from the protein A bead after binding. In addition, trypsin digestion efficiency is another challenge to consider due to lower pH after acid dissociation. Other matrix interference from endogenous immunoglobulins and etc. is another factor to carefully evaluate from this conventional trypsin digestion after acid dissociation followed by elution. Our approach, using on-bead digestion with trypsin, could have some disadvantages such as a large excess of tryptic peptide matrices derived from the protein A ligand, which might interfere with our assay. However, this method is very fast and robust and the interference from these endogenous matrix peptides could be minimized by selecting a specific signature peptide as well as its unique parent ion-product ion transition combination in the next section.

#### 3.1.2. Selection of Target-Specific Signature Peptide

A couple of specific signature peptides were sought that would be applicable and specific to adalimumab using freeware software ‘Skyline’ (MacCoss Lab Software, https://skyline.ms/). As a result, three peptides out of various digested peptides were selected from the complementarity-determining region (CDR) of adalimumab. The three specific peptide sequences are as follows: NYLAWYQQKPGK, APYTFGQGTK and GLEWVSAITWNSGHIDYADSVEGR.

**Figure 1:**
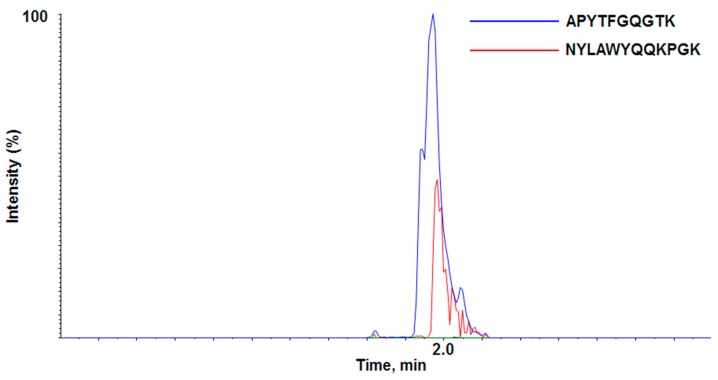
LC-QTOF-MS/MS chromatogram of the two proposed signature peptides.

During the LC-QTOF-MS/MS analysis, two out of three peptides were detected (APYTFGQGTK and NYLAWYQQKPGK), while the GLEWVSAITWNSGHIDYADSVEGR peptide was not, possibly due to poor ionization. Although both APYTFGQGTK and NYLAWYQQKPGK peptides looks acceptable, APYTFGQGTK showed better peak intensity and better signal-to-noise ratio than NYLAWYQQKPGK in our experimental conditions (Figure 1). APYTFGQGTK was not detected in rat blank plasma or any other source during sample preparation and therefore was proven to be quite a unique signature peptide representing adalimumab. Therefore, APYTFGQGTK was chosen as the specific signature peptide for the quantitation of adalimumab in our experiment.

**Figure 2:**
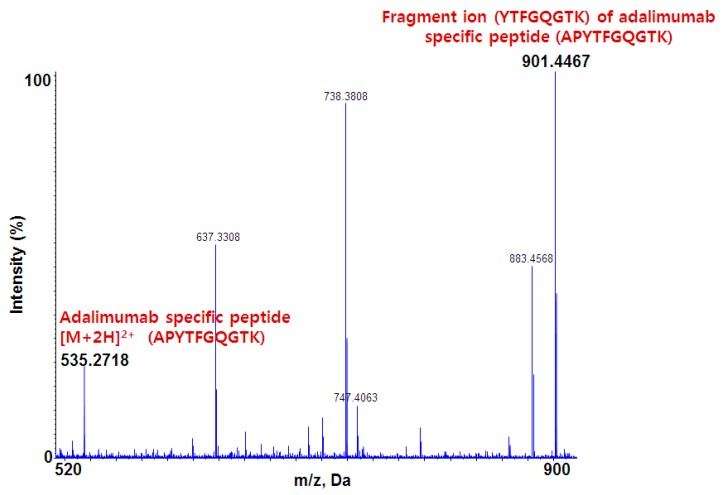
MS/MS spectrum of adalimumab specific peptide [M + 2H]^2+^ and its fragment peptide.

Figure 2 shows the MS/MS spectrum of the adalimumab-specific signature peptide (APYTFGQGTK). From the doubly charged parent ion observed at *m*/*z* 535.27, several product ions were observed and the ion at *m*/*z* 901.45 showed the best sensitivity and selectivity.

#### 3.1.3. Liquid Chromatography–Mass Spectrometry Analysis Using Quadrupole Time-of-Flight mass spectrometer

Conventionally, triple quadrupole mass spectrometers (low-resolution instrument) have been used for the quantitative analysis of small molecules. Recently, a Q-TOF mass spectrometer has been introduced to quantify large molecules such as proteins for pharmacokinetic studies [16]. In the past, Q-TOF had drawbacks in terms of sensitivity, linear dynamic range and speed, which meant it was unlikely to fill the need for reliable quantification in bioanalysis when compared to triple quadrupole mass spectrometer. However, the latest Q-TOF is good enough to overcome these shortcomings and shows good sensitivity and selectivity for the analysis of large molecules or peptides in single injection. In this study, the lower limit of quantification (LLOQ), accuracy, precision and linear dynamic range using LC-QTOF-MS/MS were good enough for our discovery non-GLP PK studies in rats.

### 3.2. Method Qualification

#### 3.2.1. Calibration Curve, Linearity and Sensitivity

**Figure 3:**
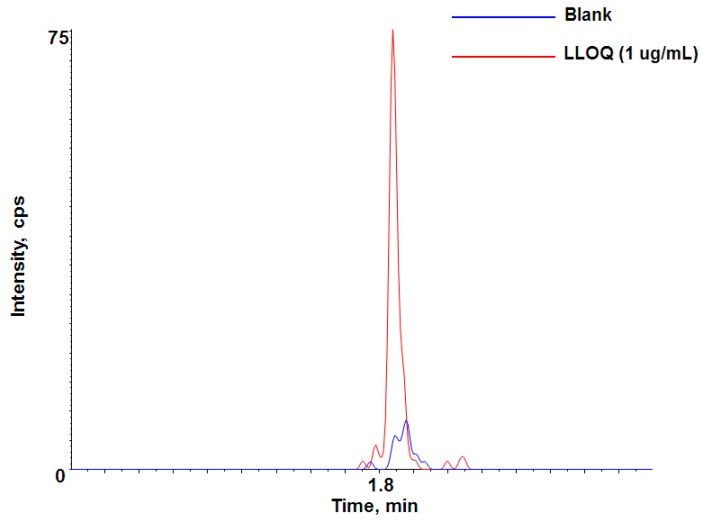
Representative chromatogram of lower limit of quantification (LLOQ, 1 µg/mL) for adalimumab-specific signature peptide in rat plasma.

LC-QTOF-MS/MS analysis using the specific signature peptide in high-resolution mode was used for the quantitation of adalimumab. Calibration curves consisting of eight points in duplicate were prepared fresh for all datasets in rat plasma. The calibration curve range was 1–100 μg/mL. A representative chromatogram at the LLOQ (signal-to-noise ratio: ~9) is shown in Figure 3. This sensitivity was significant enough for most preclinical studies with a dose level ≥1 mg/kg to cover the expected concentration range throughout the PK time course. With a larger volume (~40 µL) of sample (or trypsin), more optimized conditions of LC-QTOF-MS/MS or a more sensitive mass spectrometer, we were also able to improve the LLOQ below 1 μg/mL in the preclinical species plasma, if needed (data not shown). The quadratic regression of the curves using peak area versus concentrations was weighted by 1/concentration. The calculated correlation coefficient value (r) for calibration curve was used to evaluate the linearity of the curve. The correlation coefficient of the calibration curve was ≥0.9931 and the LLOQ of 1 μg/mL was easily achieved in this method.

#### 3.2.2. Accuracy, Precision and Species-Dependent Matrix Effect

Assay performance was determined by assessing inter/intra-day accuracy (%) and precision (%CV) of the QC samples (Table 1). Inter/intra-day accuracy and precision for rat plasma were performed using QC samples (*n* = 9) at low, medium and high QC levels. Accuracy (%) was defined as the calculated concentration, expressed as a percent deviation from nominal concentration. Precision was expressed as the percent coefficient of variation (%CV). The qualification run met the acceptance criteria of ±25% accuracy and precision for all QC samples.

**Figure 4:**
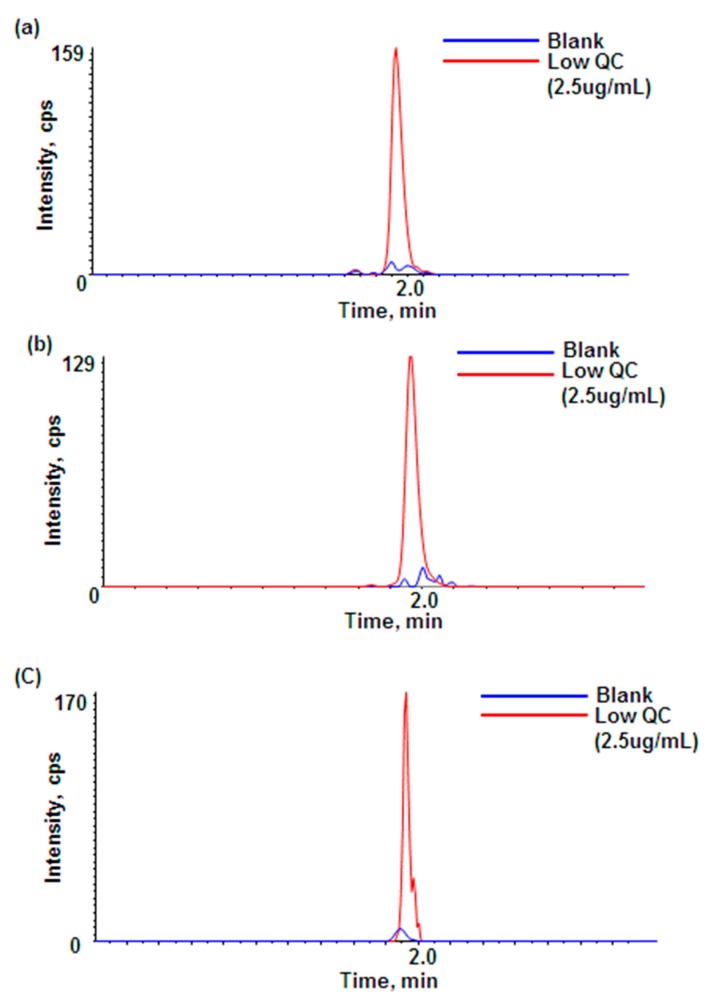
Comparison of peak intensities between blank and QC low in plasma: (**a**) blank and QC low in rat plasma; (**b**) blank and QC low in mouse plasma; (**c**) blank and QC low in monkey plasma.

This assay developed for rat plasma samples was also evaluated for plasma samples from other species (mouse and monkey). If there are no/few species-dependent matrix effects between species, the rat plasma calibration curve should be able to quantitate adalimumab in other preclinical species as well. Table 2 shows no significant species-dependent matrix effects between rat plasma and other species. Also, Figure 4 shows that the selected signature peptides are unique in the plasma of several preclinical species. Although all three levels of the QC samples were passed, the interference from the blank monkey plasma appeared to be slightly higher than in rat or mouse blank plasma. Therefore, if this method was to be applied to monkey plasma PK samples, a slightly higher LLOQ (e.g., 2 μg/mL) would be helpful for the calibration curve in monkey plasma. Overall, the rat plasma calibration curve should be applicable to analyze adalimumab in mouse and monkey plasma samples if needed.

#### 3.2.3. Freeze and Thaw Stability

Freeze and thaw stability assessments were carried out to demonstrate that adalimumab in plasma samples was stable under freeze and thaw conditions. The freeze and thaw stability experiments for rat, mouse and monkey plasma were performed using QC samples (*n* = 3) at low, medium and high QC levels. The mean values of the freeze and thaw stability QC samples at each level were compared with the nominal concentrations. The results are summarized in Table 3. The acceptance criterion for the freeze and thaw stability samples was within ± 25% precision and accuracy, which is acceptable for early drug discovery studies, and the results all met the acceptance criteria. As a result, adalimumab in rat, mouse and monkey plasma QC samples was stable through three freeze and thaw cycles.

#### 3.2.4. Application to a Pharmacokinetic Study in Rats

**Figure 5:**
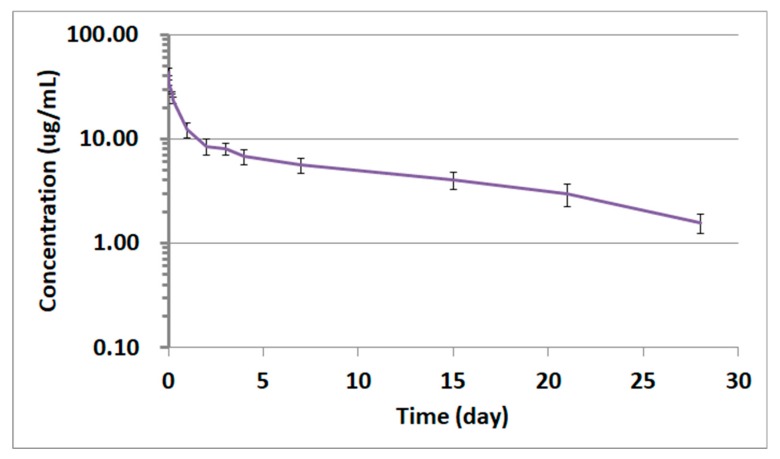
Time-concentration profile of adalimumab in rat plasma from the study subjects receiving 1 mg/kg adalimumab by intravenous administration. The data represent the mean ± standard deviation (SD, *n* = 4).

The qualified LC-QTOF-MS/MS method was successfully applied to a pharmacokinetic study of adalimumab in Sprague–Dawley (SD) rats. Plasma samples obtained after intravenous administration of 1 mg/kg were analyzed by LC-QTOF-MS/MS for the quantification of adalimumab concentrations. To assure acceptance of study sample analytical runs, at least two-thirds of the QC samples had to be within ±25% accuracy, with at least half of the QC samples at each concentration meeting these criteria. When 1 mg/kg of adalimumab was administered to the rats, the drug concentrations in rat plasma were all within the calibration curve range. The time–concentration profile is shown in Figure 5. Although no head-to-head comparison for the PK profiles was carried out between this LC-QTOF-MS/MS method and other conventional methods, the PK profile produced by LC-QTOF-MS/MS looked comparable to the reference adalimumab human PK profile published in studies using conventional methods. Unlike the PK profiles of small molecules, most monoclonal antibody drugs typically show bi-phase elimination PK profiles, which consist of a short half-life alpha-phase distribution followed by a beta-phase elimination with a long half-life. Therefore, a two-compartment model was used for the PK parameters of adalimumab in this experiment. (Table 4) [17]. The time-concentration graph of adalimumab in Figure 5 also showed typical two-compartment monoclonal antibody PK characteristics with a short alpha-phase and a long beta-phase half-life (~10 days).

## 4. Discussion

Immunoassay has been used for over 50 years and has traditionally been used to quantify large molecules. However, this method has some disadvantages [18]. The main disadvantages are that it is time-consuming and requires labor-intensive reagent development [8]. Immunoassays also often show cross-reaction with precursors of the target protein or with smaller metabolized fragments that are not suitable for early-stage mAbs bioanalysis [11]. LC-MS/MS can overcome the disadvantages of immunoassay. Compared to traditional immunoassay, LC-MS/MS technique does not require the preparation of time-consuming and high-cost reagents for specific antibody detection. Combined with LC-MS/MS and immunocapture methods, analytes can be selectively extracted without interferences such as anti-drug antibodies (ADA), which interfere with protein drug targets in the biological matrix [19].

Adalimumab is a recombinant human IgG1 monoclonal antibody [20]. Therefore, human IgG1 is also detected when quantifying adalimumab in the human matrix, making it difficult to distinguish it from the target drug. Therefore, the specific signature peptide of adalimumab was selected because it was distinguishable from human IgG1.

A LC-QTOF-MS/MS method was developed for the determination of adalimumab in rat plasma using a specific signature peptide (APYTFGQGTK) to demonstrate the feasibility of this method for adalimumab or its biosimilars. The calibration curve was acceptable over a concentration range from 1 to 100 μg/mL for adalimumab using quadratic regression with 1/concentration weighting. This LC-QTOF-MS/MS method was sensitive, selective, accurate and reproducible for the determination of adalimumab concentration and has been applied successfully to an adalimumab rat PK study. There was no significant species-dependent matrix effect between rats and other preclinical species, which means this method would be applicable to other preclinical sample analyses without developing new reagents for sample preparation. In conclusion, this method was useful for the analysis of adalimumab and could also be used as a complementary method for adalimumab or its biosimilars in early-stage research and development.

## Figures and Tables

**Table 1:** Inter/intra-day accuracy and precision of adalimumab in quality control samples.

Run No.	Statistics	QC Low	QC Med	QC High
		(2.5 µg/mL)	(25 µg/mL)	(50 µg/mL)
1	Mean	2.53	25.3	52.3
	Precision (%CV)	13.1	1.76	3.54
	*n*	3	3	3
	Accuracy (%)	101	101	105
2	Mean	2.73	23.9	56.3
	Precision (%CV)	2.6	11.13	11.55
	*n*	3	3	3
	Accuracy (%)	109	95	113
3	Mean	2.57	25.1	47.3
	Precision (%CV)	17.3	10.54	4.44
	*n*	3	3	3
	Accuracy (%)	103	100	95
Inter-day	Mean	2.61	24.77	51.97
	Precision (%CV)	11	7.81	6.51
	*n*	9	9	9
	Accuracy (%)	104	99	104

**Table 2:** Various species-dependent matrix effects in mouse and monkey plasma. (a) Quality control result in mouse plasma; (b) quality control result in monkey plasma.

(**a**)					
**Mouse**	**Theoretical Concentration (μg/mL)**	**Mean Concentration (μg/mL)**	**Precision** **(%CV)**	***n***	**Accuracy (%)**
QC low	2.5	2.85	13.2	3	114
QC medium	25	29.4	5.74	3	118
QC high	50	57	1.43	3	114
(**b**)					
**Monkey**	**Theoretical Concentration (μg/mL)**	**Mean Concentration (μg/mL)**	**Precision** **(%CV)**	***n***	**Accuracy (%)**
QC low	2.5	2.09	4.9	3	84
QC medium	25	23.4	11.19	3	94
QC high	50	48.1	5.12	3	96

**Table 3:** Freeze and thaw stability assessment in preclinical species (three cycles) (**a**) Freeze and thaw stability in rat plasma; (**b**) freeze and thaw stability in mouse plasma; (**c**) freeze and thaw stability in monkey plasma.

(**a**)					
**Rat**	**Theoretical Concentration (μg/mL** **)**	**Mean Concentration (μg/mL** **)**	**Precision (%)**	***n***	**Accuracy (%)**
QC low	2.5	2.58	10.5	3	103
QC medium	25	25.6	13.67	3	102
QC high	50	57.3	8.73	3	115
(**b**)					
**Mouse**	**Theoretical Concentration (μg/mL** **)**	**Mean Concentration (μg/mL** **)**	**Precision (%)**	***n***	**Accuracy (%)**
QC low	2.5	2.62	16.7	3	105
QC medium	25	28	3.26	3	112
QC high	50	60.2	0.94	3	120
(**c**)					
**Monkey**	**Theoretical Concentration (μg/mL** **)**	**Mean Concentration (μg/mL** **)**	**Precision (%)**	***n***	**Accuracy (%)**
QC low	2.5	2.26	2.8	3	91
QC medium	25	19.5	12.42	3	78
QC high	50	40.3	8.52	3	81

**Table 4:** Pharmacokinetic parameters of adalimumab after 1 mg/kg intravenous injection in rats.

PK parameters								
Compound	AUC (μg∙Day/mL)	Cl (mL/Day/kg)	Alpha Half Life (Day)	Beta Half Life (Day)	C_max_ (μg/mL)	V_1_ (mL/kg)	V_ss_ (mL/kg)	Compartment Model
**Adalimumab**	155.29	6.68	0.2	9.82	41.64	24.1	85.83	2
